# Cornual Heterotopic Pregnancy after Bilateral Salpingectomy and Uterine Septum Resection Resulting in Term Delivery of a Healthy Infant

**DOI:** 10.1155/2014/157030

**Published:** 2014-11-05

**Authors:** Serkan Oral, Yaşam Kemal Akpak, Nilay Karaca, Ali Babacan, Kadir Savan

**Affiliations:** ^1^Department of Assisted Reproduction, Liv Hospital, 34340 Istanbul, Turkey; ^2^Department of Obstetrics and Gynecology, Ankara Mevki Military Hospital, 06100 Ankara, Turkey; ^3^Department of Assisted Reproduction, 29 Mayis Hospital, 34250 Istanbul, Turkey; ^4^Department of Obstetrics and Gynecology, Gulhane Military Medical Academy, Haydarpasa Training Hospital, 34668 Istanbul, Turkey; ^5^Department of Assisted Reproduction, Medical Park Bahcelievler Hospital, 34160 Istanbul, Turkey

## Abstract

Heterotopic pregnancy is the simultaneous occurrence of two or more implantation sites. A 25-year-old infertile patient with a history of bilateral salpingectomy, uterine septum resection, and left cornual resection was diagnosed with heterotopic pregnancy in her second *in vitro* fertilization trial. She attended our clinic when she was 7-week pregnant, complaining initially of severe abdominal pain. Findings associated with peritoneal irritation were positive during the physical examination. Transvaginal ultrasound revealed right cornual ectopic pregnancy with a live fetus in the middle of the uterine cavity. Also free fluid was noted in the pelvis. A diagnosis of heterotopic pregnancy with rupture of the cornual pregnancy was made. She underwent emergency laparoscopy with aspiration of the ruptured ectopic pregnancy, suturing to the entire visible cornual margins, and assurance of good haemostasis. Her recovery was uneventful and she continued receiving care in our obstetric unit. She delivered a healthy newborn by cesarean section at term.

## 1. Introduction

Ectopic pregnancy is a common and potentially hazardous condition which refers to the implantation of a viable ovum elsewhere other than the uterine corpus. Heterotopic pregnancy (HP) is a multiple pregnancy, with one embryo being viably implanted in the uterus and the other(s) being implanted outside the uterine corpus as an ectopic pregnancy. It is most often represented as intrauterine pregnancy in the presence of ectopic pregnancy [1]. Heterotopic pregnancies are extremely rare in the general population, especially in spontaneous pregnancies. However, after the extensive use of parenteral gonadotropin therapy in assisted reproduction techniques (ART), the incidence of HP is approximately between 1 : 7963 and 1 : 30.000 in the general population [2]. In pregnancies resulting from assisted reproduction techniques, the incidence ranges from 1/100 to 1/3,600 [3]. A cornual ectopic pregnancy is one of the most life-threatening types of ectopic gestations, which accounts for 2–4% of all the ectopic pregnancies, and it has a mortality rate which is 6-7 times higher than that of the ectopic pregnancies in general [4]. Early diagnosis of interstitial ectopic pregnancies is pivotal, as they often remain asymptomatic until rupture occurs and carries a mortality risk. Term delivery of a healthy infant after rupture of a heterotopic cornual pregnancy is extremely rare. Therefore, we report a case with delivery to term of a healthy infant following the rupture of a heterotopic cornual pregnancy.

## 2. Case Report

**Figure 1:**
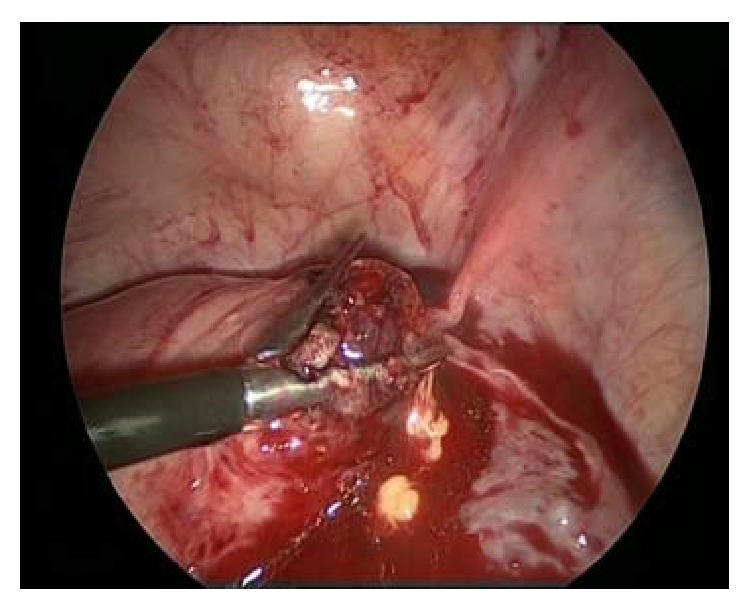
Laparoscopic findings showing a ruptured right uterine cornual ectopic pregnancy with hemoperitoneum.

**Figure 2:**
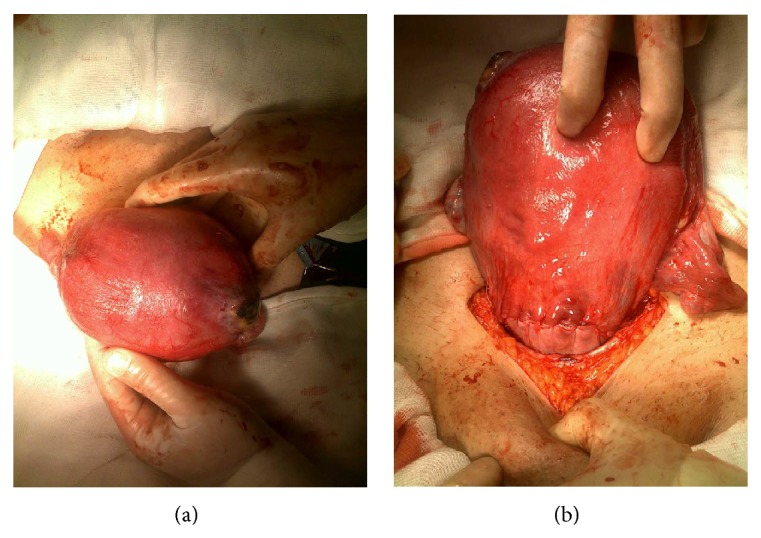
Stumps of right and left uterine cornua after a successful operation of caesarean section.

A 27-year-old woman underwent her second* in vitro* fertilization (IVF) procedure for tubal factor infertility. Previously, she had bilateral salpingectomy and hysteroscopic septum resection due to bilateral hydrosalpinx and uterine septum before IVF trial. She had a laparoscopic left cornual resection due to cornual pregnancy in her fist IVF trial. Two embryos were transferred in her second IVF cycle. In the first examination of the patient, a gestational sac was visualized in the uterus. However, the second cornual gestational sac was not observed. The patient was admitted with a complaint of sudden onset of severe abdominal pain in the seventh week of pregnancy. Physical examination revealed muscular defense and rebound tenderness. Free echogenic pelvic fluid correlation with hemoperitoneum was identified by transvaginal sonography. An ultrasound scan showed an intrauterine gestational sac with a viable embryo and a hypoechoic region in the right cornual part of the uterus. The patient's hemoglobin concentration was 7.5 g/dL. An emergency laparoscopy was performed. Operation revealed an extensive hemoperitoneum and ruptured right uterine cornua (Figure 1). The cornual pregnancy was aspirated and the hemostasis was obtained by bipolar electrocoagulation with great caution in order not to damage intrauterine gestation. The entire visible cornual margins were reapproximated with one layer of 0 polyglactin 910 (Vicryl, Ethicon Inc., Somerville, NJ). Blood transfusion was commenced during surgery and she was transfused with 2 units of blood. The postoperative course was uneventful, viability of the intrauterine pregnancy was confirmed postoperatively, and she was discharged on her second postoperative day in a hemodynamically stable condition. The intrauterine pregnancy proceeded without any complications. A full-term 3200-gram male infant was delivered by primary cesarean section, which was performed owing to her prior corneal resection (Figure 2).

## 3. Discussion

The frequency of heterotopic pregnancy, though still uncommon, has increased after ART [1–3]. Cornual pregnancy is a rare subtype of ectopic pregnancy. Owing to its unique location, the diagnosis and treatment could be difficult [4].

Different mechanisms could predispose the development of HP after IVF cycles. This increased risk is associated with implantation of more than one embryo and tubal damage caused by, for example, previous tubal ectopic pregnancy, pelvic inflammatory disease, and tubal malformation in patients with infertility [1].

HP is potentially fatal and its diagnosis is a major predicament for clinicians. Clinical features can vary widely, with some patients being asymptomatic and others experiencing severe abdominal pain and presenting before or in hypovolemic shock. Transvaginal sonographic (TVS) assessment is essential for accurate diagnosis of HP. When intrauterine pregnancy (IUP) is detected, clinicians must not disclaim the possibility of coexistence with extrauterine pregnancy in above-described patients. Using Doppler ultrasound can improve the chance of diagnosing [2].

Treatment options of HP are surgery, medical treatment, and expectant management for maintaining IUP. Medical treatment, with potassium chloride or hyperosmolar injection directly in the ectopic pregnancy using an ultrasound-guided procedure, and surgical treatments have similar intrauterine gestation outcomes [5]. Methotrexate is usually avoided due to the risk of toxicity for the intrauterine pregnancy [5]. Nearly a third of women with HP present with hemodynamic instability because of rupture. Therefore, laparotomy could be the first option with these patients [3]. Laparoscopic suturing is a controversial issue in laparoscopic surgery for cornual pregnancies [6]. If a myometrial gap results from evacuation of the cornual pregnancy, the gap requires suture closure to minimize the risk of uterine rupture with IUP [6]. In the present case we found using suture appropriate.
